# Self-reported functional status predicts post-operative outcomes in non-cardiac surgery patients with pulmonary hypertension

**DOI:** 10.1371/journal.pone.0201914

**Published:** 2018-08-16

**Authors:** Aalap C. Shah, Kevin Ma, David Faraoni, Daniel C. S. Oh, G. Alec Rooke, Gail A. Van Norman

**Affiliations:** 1 Department of Anesthesiology and Pain Medicine, University of Washington Medical Center, Seattle, Washington, United States of America; 2 Department of Anesthesia and Pain Medicine, Hospital for Sick Children, University of Toronto, Toronto, Ontario, Canada; 3 School of Medicine, University of Washington, Seattle, Washington, United States of America; University of Bern, University Hospital Bern, SWITZERLAND

## Abstract

**Background:**

Pulmonary hypertension (PHTN) is associated with increased post-procedure morbidity and mortality. Pre-procedure echocardiography (ECHO) is a widely used tool for evaluation of these patients, but its accuracy in predicting post-procedure outcomes is unproven. Self-reported exercise tolerance has not been evaluated for operative risk stratification of PHTN patients.

**Objective:**

We analyzed whether self-reported exercise tolerance predicts outcomes (hospital length-of-stay [LOS], mortality and morbidity) in PHTN patients (WHO Class I–V) undergoing anesthesia and surgery.

**Methods and findings:**

We reviewed 550 non-cardiac, non-obstetric procedures performed on 370 PHTN patients at a single institution between 2007 and 2013. All patients had cardiac ECHO documented within 1 year prior to the procedure. Pre-procedure comorbidities and ECHO data were collected. Functional status (< or ≥ 4 metabolic equivalents of task [METs]) was assigned based on responses to standard patient interview questions during the pre-anesthesia clinic visit. Multiple logistic regression was used to develop a risk score model (Pulmonary Hypertension Outcome Risk Score; PHORS) and determine its value in predicting post-procedure outcomes. In an adjusted model, functional status <4 METs was independently associated with a LOS >7 days (p < .003), as were higher ASA class (p < .002), open surgical approach (p < .002), procedure duration > 2 hours (p < .001), and the absence of systemic hypertension (p = .012). PHORS Score ≥2 was associated with an increased 30-day major complication rate (28.7% vs. 19.2%; p < 0.001) and ICU admission rate (8.6% s 2.8%; p = .007), but no statistical difference in hospital readmissions rate (17.6% vs. 14.0%; p = .29), or mortality (3.5% vs. 1.4%; p = .75). Similar ECHO findings did not further improve outcome prediction.

**Conclusions:**

Poor functional status is associated with severe PHTN and predicts increased LOS and post-procedure complications in patients with moderate to severe pulmonary hypertension with different etiologies. A risk assessment model predicts increased LOS with fair accuracy. A thorough evaluation of underlying etiologies of PHTN should be undertaken in every patient.

## Introduction

Patients with significant pulmonary hypertension (PHTN) are at elevated risk for post-procedure complications [1–5] and are presenting for elective anesthesia and non-cardiac surgery (NCS) with increasing frequency. Previous studies have reported increased perioperative morbidity, mortality, and hospital length-of-stay (LOS) in PHTN patients undergoing non-cardiac and non-obstetric surgery, [1,5,6] although no distinction has been made between mildly, moderately or severely affected patients. Right ventricular (RV) failure and persistent post-procedure hypoxia requiring mechanical ventilator support are among the most frequently reported major post-procedure complications, with a cited incidence >10%. [1,2,7–9] However, existing studies have lacked sufficient size and power to decisively evaluate PHTN class or severity, related cardiac dysfunction, or surgery and anesthesia approach as predictors of adverse post-procedure outcomes. [4,10]

Gold standards for routine evaluation of PHTN patients (echocardiography [ECHO] and/or cardiac catheterization [1] are expensive [11], and repeat pre-operative testing in patients with recent ECHO may yield little additional value in risk stratification and inevitably delay the procedure. Mean pulmonary artery pressure and pulmonary vascular resistance do not appear to predict outcomes following NCS [12], while some studies suggest that ECHO findings such as higher pulmonary artery systolic pressure (PASP), a ratio of pulmonary systolic to systemic systolic pressure ≥0.66 and the presence of RV hypertrophy may be somewhat predictive of worse post-procedure course. [10,13,14]

Recent efforts have focused on the evaluation of functional status (FS) as a surrogate measure to stratify PHTN severity and for risk categorization [4,15–18] for post-procedure complications. However, ordinal systems such as the World Health Organization (WHO) and New York Heart Association (NYHA) are associated with low inter-rater reliability. [17,19] Previous studies have, however, demonstrated an association between poor FS and an increased risk of post-procedure complications in patients without PHTN. [20–22] Experience in our pre-anesthesia clinics suggested that patients with a self-reported exercise tolerance ≥ 4 metabolic equivalents of task (METs) rarely have severe PHTN (PASP ≥59 mmHg) [23] or concurrent RV dysfunction. However, this experience had never been confirmed by formal study, nor has it been correlated with outcomes.

We sought to determine if a correlation exists between self-reported exercise tolerance in patients who have a previously established cardiac catheterization diagnosis of PHTN and PHTN severity, longer post-procedure LOS, and/or other post-procedure outcome measures. Because of our current pre-anesthesia risk stratification protocol, we evaluated all procedures in patients with any PHTN etiology (WHO Class I–V). In addition, we identified factors that independently predict adverse post-procedure outcomes, and constructed a predictive risk scoring system for patients with PHTN using those variables and FS.

## Methods

A retrospective cohort study was conducted with Institutional Review Board approval from the University of Washington after waiver of the requirement for informed consent. We initially reviewed patient charts of 1922 adult (age ≥18) patients with a prior cardiac catheterization diagnosis of PHTN (mean pulmonary artery pressure ≥ 25 mmHg at rest and a pulmonary capillary wedge pressure ≤ 15 mmHg) who received general anesthesia for elective surgery at our institution from April 2007 to September 2013. All data were fully anonymized prior to retrieval. Inclusion criteria consisted of 1) a diagnosis of PHTN of any class or severity, based on ECHO or cardiac catheterization; 2) available resting ECHO data within 1 year prior to scheduled procedure; and 3) elective non-cardiopulmonary bypass, non-obstetric procedures associated with general or regional anesthesia, deep sedation, or monitored anesthesia care. We included both same-day admit patients as well as inpatients awaiting surgery in the study. When patients had undergone multiple procedures during a single hospitalization, we included data only from the initial procedure. Cases were excluded if 1) the diagnosis of PHTN was made after the procedure or surgery of interest; 2) the patient was admitted to an inpatient ward or ICU bed >24 hours prior to the procedure; 3) there was absent or incomplete pre-procedure anesthesia clinic visit data, including patient-reported FS; or 4) the procedure or surgery was cancelled prior to or after administration of anesthesia.

### Patient demographics and comorbidities

Comorbidities that were recorded included significant cardiovascular disease (previous myocardial infarction, arrhythmias, congestive heart failure (CHF), pulmonary disease (asthma, chronic obstructive pulmonary disease, diagnosis of or 3 risk factors for obstructive sleep apnea, smoking history), history of deep venous thrombosis, diabetes, or chronic renal disease. Pre-procedure clinical weight and body mass index, anticipated post-procedure disposition of the patient (outpatient vs. inpatient) and the number of previous procedures and surgeries were obtained from the pre-anesthesia clinic note. World Health Organization (WHO) classification of PHTN subtypes was determined by a review of cardiac catheterization report data and consultant notes related to the procedure and underlying diagnoses specific to each class. Post-capillary PHTN (i.e. WHO Class II) was determined by a pulmonary capillary wedge pressure (PCWP) ≥ 15 mmHg. (24)

### Self-reported exercise tolerance

During the pre-anesthesia clinic visit, all patients were asked to estimate the number of blocks they could walk and flights of stairs they could climb without experiencing symptomatic limitation. This information was used to group patients based on their METs, a measure of FS. Patients who could not walk 4 blocks and/or climb 2 flights of stairs were considered to have poor exercise tolerance (FS <4 METs). [1,20,25–27].

### Two-dimensional ECHO

Two-dimensional pre-procedure echocardiogram (ECHO) data performed within 1 year of the index surgical date were recorded. If multiple ECHO scans were performed, the study closest to the surgical date was used for analysis.

### Outcome measures

Primary outcomes were hospital length-of-stay (LOS), death, unplanned ICU admission, hospital LOS, and hospital readmission within 30 days of surgery. Secondary outcomes included the incidence of post-procedure complications within 30 days of the procedure. Outcomes were determined from the review of hospital progress notes, post-anesthesia care unit (PACU) records, discharge summary note, and follow-up outpatient notes. Hospital length of stay (LOS) was defined as the interval from the date of surgery until date of hospital discharge or in-hospital death. Hospital readmission within 30 days of the procedure was obtained from admission notes in the electronic medical record.

### Statistical analysis and internal validation of risk score system

The Shapiro-Wilk normality test was used to assess continuous variables for normality. Continuous variables are expressed as mean and standard derivation, and categorical variables are expressed as number and percentage (%). Univariable logistic regression analysis was performed to identify all possible determinants for prolonged LOS (> 7 days). To control for possible confounding among variables, we used multivariable logistic regression to determine the independent predictors using a univariable p-value cut off of < 0.10 for inclusion. Multivariate analyses were adjusted for cardiopulmonary comorbidities (e.g. coronary artery disease [CAD], congestive heart failure [CHF], chronic obstructive pulmonary disease [COPD]), venous thromboembolic (VTE]) disease, and serum creatinine > 1.5 mg/dl. Cut off values for continuous variables were determined using receiver operating characteristic (ROC) curve analysis and the Youden J-index. The results are expressed as regression coefficient (B) and standard error (SE), the odds ratio (OR) as a measure of risk, the 95% confidence interval (CI), and p-values obtained from the Wald test. Generalized estimating equations (GEE) [28] were used for both uni- and multivariable analysis to adjust for patients having multiple hospital admissions during the study period. Area under the receiver operating characteristics curve (AUC) was used to assess model performance.

Based on the predictors obtained from multivariable logistic regression, we developed a simple score to predict the incidence of prolonged LOS >7 days (Pulmonary Hypertension Outcomes Risk Score; PHORS). Each risk factor was weighted as 1 point, and the simple sum was defined as the final PHORS value. Subsequently, patients were dichotomized according to their risk score (< 2 or ≥ 2), and the incidence of complications was compared between the 2 groups. We used the study cohort to evaluate the performance of the risk score for predicting mortality. Performance was evaluated by assessing calibration, assessed graphically by plotting the observed frequency of mortality against the predicted in-hospital probability for mortality. A smooth, nonparametric calibration line was created using the locally weighted scatterplot-smoothing (LOESS) [29] algorithm to estimate the observed probability of mortality in relation to the predicted probabilities. Discrimination was calculated using the concordance statistic (*c*-statistic) and the Brier score.

All p-values were two-tailed, and a p-value < 0.05 was considered significant.

Statistical analysis was performed using STATA (version 14.1 for Mac OS, Stata Corp, College Station, TX).

## Results

### Study population

Of 1922 potential procedures, 661 cases were deemed eligible, based on fulfillment of all 3 inclusion criteria. Of these procedures, 34 cases were excluded as secondary procedures occurring during the same hospitalization, 43 cases were excluded due to missing data regarding functional status, 31 cases were excluded as the patient was admitted ≥24 hours prior to the procedure date, 3 cases were excluded because the procedure was abandoned after anesthesia administration. After exclusions, 550 cases involving 370 patients were included for analysis. Data regarding PHTN severity (PASP) were available in 527 cases. Case characteristics including demographics and comorbidities are summarized in Table 1.

**Table 1:** Demographic characteristics of patients with PHTN by PHTN severity category.

Characteristics	Mild PHTN (PASP 25–40 mmHg) (n = 230)	Moderate PHTN (PASP 41–59 mmHg) (n = 247)	Severe PHTN (PASP > 59 mmHg) (n = 50)	p-value^¶^
Male gender (%)	125 (54)	130 (53)	23 (46)	.37
Age (years) (mean [SD])	59 (14)	62 (13)	59 (16)	.60
Body mass (kg) (mean [SD])	90 (29)	93 (33)	96 (56)	.58
Height (cm) (mean [SD])	170 (9)	170 (10)	169 (11)	.42
BMI (kg/m^2^) (mean [SD])	31 (9)	32 (12)	34(23)	.49
Anticipated inpatient post-procedure disposition (%)	154 (67)	164 (46)	37 (74)	.34
ASA classification (%)				.001
II	22 (10)	19 (8)	2 (4)	
III	162 (70)	171 (70)	25 (50)	
IV	46 (20)	57 (23)	23 (46)	
WHO Class (%)				.13
I	141 (61)	130 (53)	22 (44)	
II	48 (21)	66 (27)	14 (28)	
III	28 (12)	39 (16)	11 (22)	
IV	13 (6)	9 (3)	3 (6)	
V	0 (0)	3 (1)	0 (0)	
Post-capillary PHTN (%)	48 (38)	66 (27)	14 (28)	.268
Current tobacco use (%)	11 (5)	20 (8)	4 (8)	.76
Poor self-reported FS (< 4 METs) (%)	108 (47)	124 (50)	31 (62)	.049
Open surgical approach (%)	122 (53)	127 (51)	29 (58)	.46
Systemic hypertension (%)	150 (65)	166 (67)	33 (66)	1.00
Angina (%)	16 (7)	17 (7)	5 (10)	.39
Coronary artery disease (%)	61 (27)	92 (38)	19 (38)	.43
Congestive heart failure (%)	57 (25)	84 (34)	24 (48)	.010
Arrhythmia (%)	90 (39)	115 (47)	25 (50)	.37
Venous thromboembolism (%)	14 (6)	14 (6)	5 (10)	.23
Asthma (%)	25 (11)	40 (16)	9 (18)	.39
COPD (%)	20 (9)	40 (16)	13 (26)	.016
Obstructive sleep apnea (%)	56 (24)	67 (27)	13 (26)	1.00
Diabetes (%)	56 (24)	76 (31)	19 (38)	.14
Renal failure (serum creatinine > 1.5 mg/dl) (%)*	34 (19)	53 (29)	9 (32)	.046
PHTN medical therapy (%)^Ŧ^	6 (3)	3 (1)	8 (16)	< .001
Number of procedures (n) (median [IQR)]	2 (1–3)	2 (1–3)	1 (1–2)	.036
1	110 (48)	117 (47)	30 (60)	
2	46 (20)	67 (27)	12 (24)	
3+	74 (14)	63 (26)	8 (16)	
Most recent procedure (%)	156 (68)	156 (63)	39 (78)	.08
Procedure length (hours) (median [IQR)]**	1.3 (0.6–2.6)	1.6 (0.6–2.7)	1.0 (0.4–2.3)	.07
**ECHO finding**				
RAP ≥ 10 mmHg***	51 (28)	88 (50)	26 (75)	< .001
PASP or RVSP (median [IQR])****	36 (32–38)	48 (43–52)	70 (60–83)	< .001
LVEF < 40%*****	67 (28)	72 (30)	16 (33)	.439

Data reported as n (%) unless otherwise specified.

^¶^ = Statistical analysis conducted for comparisons between non-severe PHTN and severe PHTN patients after adjustment for multiple admissions. Chi-squared test used for categorical data, two-tailed t-test used for normally distributed continuous data, and Mann-Whitney U test used for non-normally distributed continuous data.

^**Ŧ**^ = Therapy includes one or more of the following medications: bosentan, sildenafil, taldalafil, and ambrisentan.

* Data available in n = 181 cases (mild PHTN), n = 182 cases (moderate PHTN), n = 28 cases (severe PHTN).

** Data available in n = 229 cases (mild PHTN), n = 247 cases (moderate PHTN), n = 48 cases (severe PHTN).

*** Data available in n = 184 cases (mild PHTN), n = 176 cases (moderate PHTN), n = 34 cases (severe PHTN).

**** Data available in n = 230 cases (mild PHTN), n = 247 cases (moderate PHTN), n = 50 cases (severe PHTN).

***** Data available in n = 220 cases (mild PHTN), n = 234 cases (moderate PHTN), n = 42 cases (severe PHTN)

Abbreviations: ASA = American Society of Anesthesiologists; BMI = body mass index; COPD = chronic obstructive pulmonary disease; ECHO = echocardiography; FS = functional status; IQR = interquartile range; LVEF = left ventricular ejection fraction; PAH = pulmonary arterial hypertension; PASP = pulmonary artery systolic pressure; PHTN = pulmonary hypertension; RAP = right atrial pressure, RVSP = right ventricular systolic pressure; WHO = World Health Organization

### Self-reported exercise tolerance and ECHO findings

When categorized by self-reported exercise tolerance (FS < or ≥ 4 METs), there was no statistically significant association between the prevalence of post-capillary PHTN (e.g. WHO Class II) and FS < 4 METs. (27.8% vs. 21.7%; p = .114). Compared to patients with FS ≥ 4 METs, a significantly greater proportion of patients with elevated right atrial pressure (RAP > 10 mmHg) were represented in the FS <4 METs group (56.8% vs. 43.8%; p = .012), as were patients with left ventricular dysfunction as ascertained by reduced ejection fraction (EF) ≤ 40% (28.6% vs. 19.1%; p = .015). There was no difference with regard to the average PASP or other ECHO findings (ventricular size, thickness, RV function, valvular abnormalities) between the functional status groups. Fifty procedures were completed in patients with severe PHTN (PASP or RVSP >59 mmHg) [22]). There was no statistically significant association between FS and severe PHTN as ascertained by the PASP cutoff value (p = .076). In the subgroup of procedures undertaken in patients with pulmonary arterial hypertension (PAH; WHO Class I), there was no statistically significant association between FS < 4 METs and the severe PHTN (PASP > 59 mmHg) (59.1% vs. 41.9%; p = .12) In this same subgroup, there was a statistical association between FS < 4 METs and elevated RAP (49.4% vs. 35.5%; p = .048)

### Predictors of LOS

S1 and S2 Tables summarize case characteristics and procedure categories by LOS. LOS ≤7 days occurred in 433 cases (78.7%), and a LOS >7 days occurred in 117 (21.3%) cases. Poor self-reported exercise tolerance (FS < 4 METs), ASA class, an open surgical approach, longer procedural duration and the absence of a diagnosis of systemic hypertension were each associated with a LOS >7 days. Post-capillary pathophysiology was not statistically associated with LOS > 7 days (29% vs 24%; p = .23). There was no statistical association between LOS >7 days and median PASP (p = .15) or RAP (p = .72) (S1 Table). There were no intraoperative cardiac arrests or deaths. Patients with LOS > 7 days demonstrated a greater prevalence of intra-operative pressor use and continued post-operative ventilatory support compared to patients with LOS ≤7 days. (S3 Table) In the subgroup of procedures undertaken in patients with pulmonary arterial hypertension (PAH; WHO Class I), there was a a statistically significant association between FS < 4 METs and the probability of LOS > 7 days (63.0% vs. 39.6%; p = .002). In this same subgroup, severe PHTN based on ECHO (PASP > 59 mmHg) was not associated with LOS > 7 days (7.7% vs. 7.5%; p = 1.00).

Logistic regression analysis revealed FS < 4 METs, open surgical approach, procedural duration > 2 hours, absence of pre-existing systemic hypertension, and need for post-procedure ventilatory support as factors independently associated with LOS > 7 days. (Table 2) AUC was 0.76 (95% CI 0.71–0.81). An adjusted model demonstrated statistical significance for FS < 4 METs, open surgical approach, procedural duration > 2 hours, and the absence of pre-existing systemic hypertension. AUC was 0.77 (95% CI: 0.71–0.82).

**Table 2:** Multivariable regression analysis for predictors of prolonged LOS > 7 day.

Variables	Unadjusted model		Adjusted model	
	OR (95% CI)	p-value	OR (95% CI)	p-value
ASA classification (IV)	2.3 (1.5–3.8)	<.001	2.5 (1.4–441)	.001
Poor functional status (< 4 METs)	2.1 (1.3–3.3)	.002	2.2 (1.4–3.7)	.001
Absence of systemic hypertension	1.9 (1.2–3.0)	.008	1.9 (1.1–3.2)	.017
Open surgical approach	2.9 (1.8–4.8)	<.001	2.4 (1.3–4.0)	.003
Procedure duration (> 2 hours)	2.2 (1.4–3.5)	.001	2.5 (1.5–4.3)	.001
Post-procedure ventilatory support	2.6 (0.8–9.2)	.13	1.7 (0.4–7.1)	.434
Post-capillary PHTN	1.1 (0.7–1.8)	.71	1.0 (0.5–1.8)	.893

Data obtained from multiple logistic regression analysis after adjustment for multiple admissions, and presented as regression coefficient (B), standard error (SE), odds ratio (OR), and 95% confidence interval (CI), Wald test p-value. Analyses adjusted for age, multiple surgical procedures, coronary artery disease (CAD), congestive heart failure (CHF), asthma, chronic obstructive pulmonary disease (COPD), venous thromboembolic disease (VTE), and serum creatinine > 1.5 mg/dl.

Abbreviations: ASA = American Society of Anesthesiologists; HTN = hypertension; METS = metabolic equivalents of task; PASP = pulmonary artery systolic pressure

### PHTN Outcome Risk Score (PHORS)

**Fig 1:**
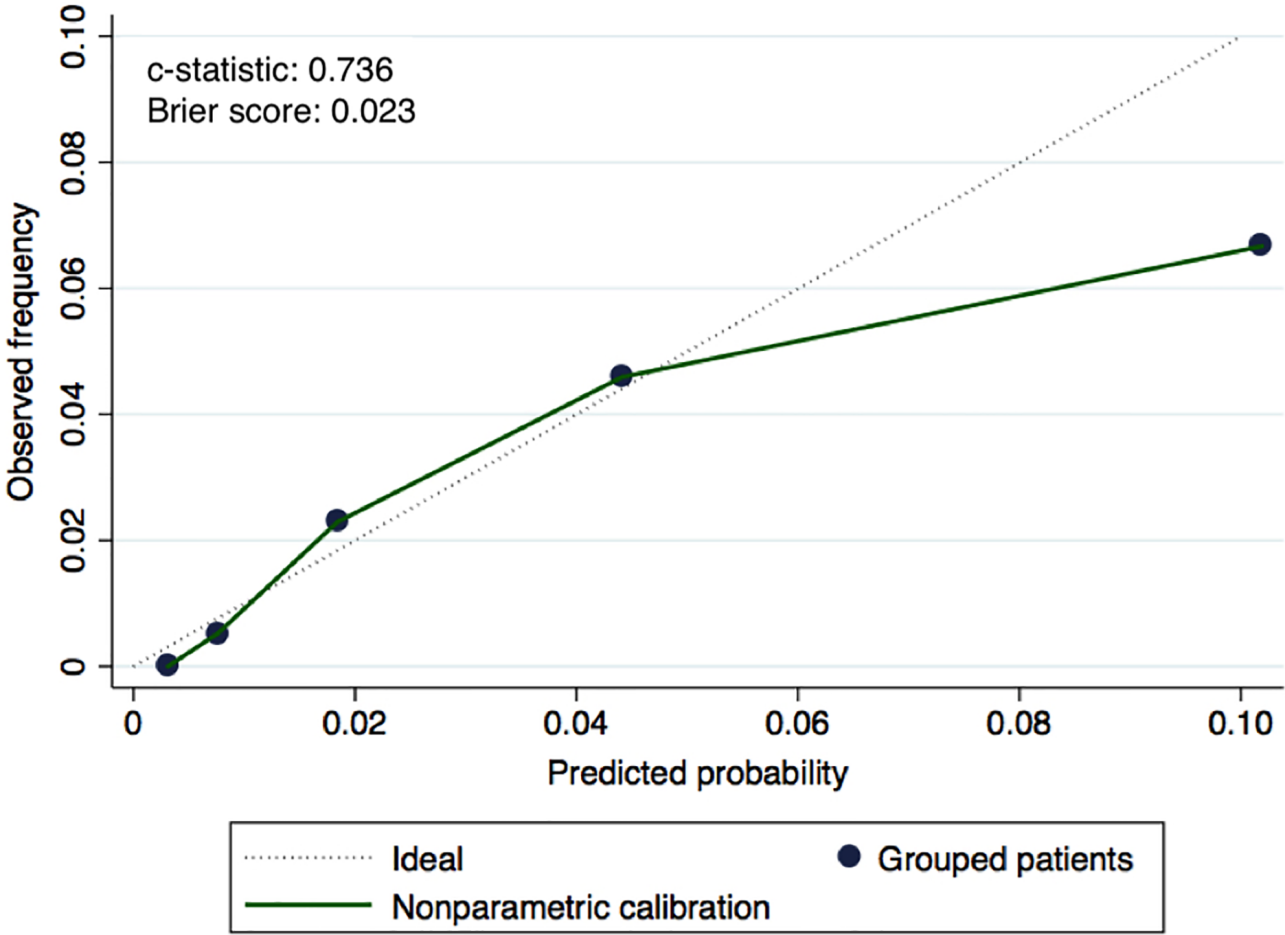
Nonparametric calibration for assessment of internal validity.

A risk scoring system (PHORS) was constructed with the significant factors identified in the adjusted binary logistic regression analysis. ASA classification has a known clinical overlap with FS and demonstrated a significant statistical association with FS (p = .003), and was not included in the PHORS model. AUC was 0.74 (95% CI: 0.70–0.80). Assessment showed good calibration (Fig 1), with a high concordance between the predicted probabilities obtained from the logistic regression and the observed frequencies using the risk stratification score. The score showed a good discrimination with a *c*-statistic of .733 and the Brier score of .023.

**Fig 2:**
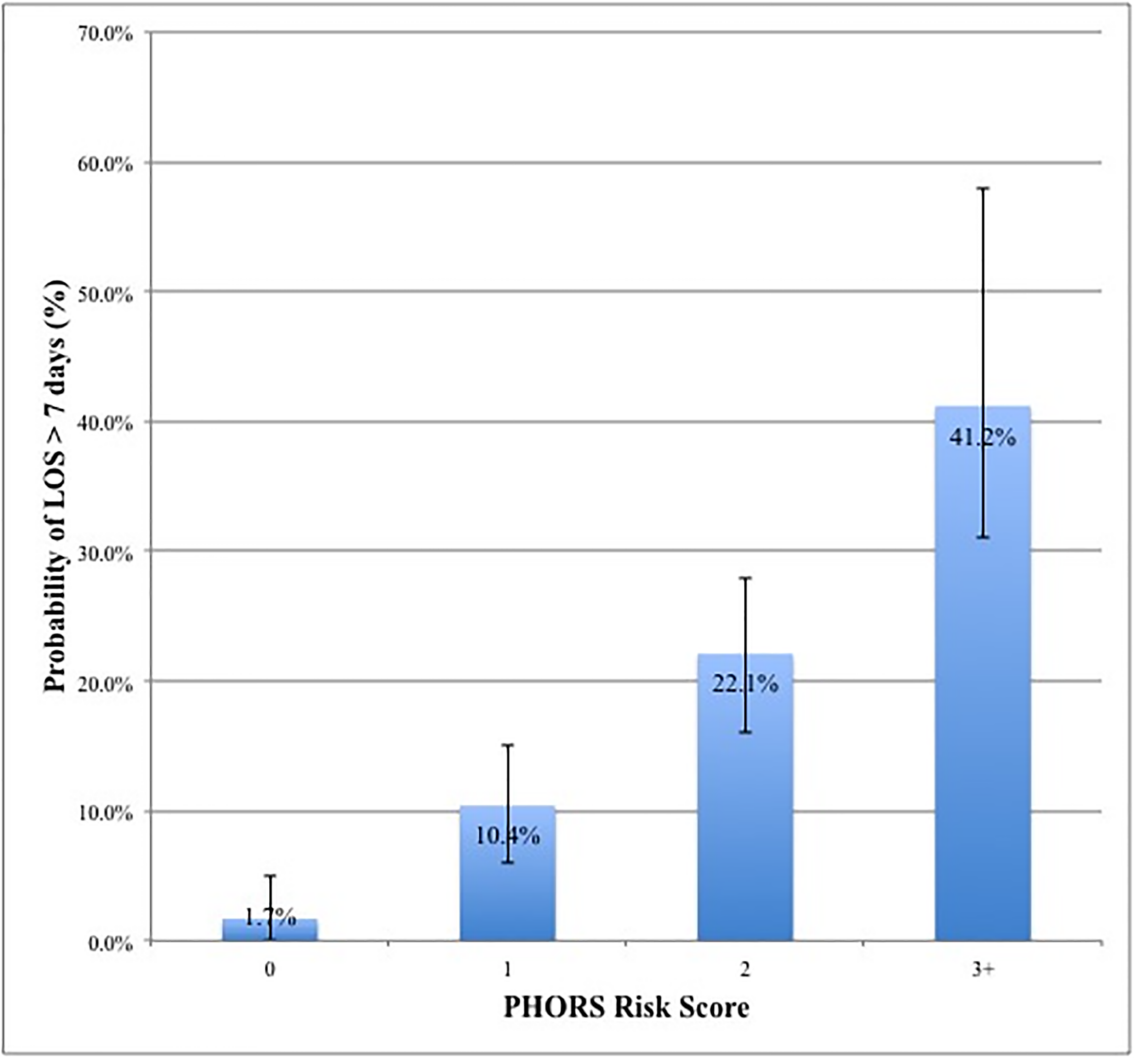
PHORS risk score and predicted probability of LOS > 7 Days.

We included severe PHTN (PASP and/or RVSP > 59 mmHg) in the final PHORS risk stratification models. AUC was 0.75 (95% CI: .70–.81). Substituting right atrial pressure (RAP) >10 mmHg for PASP resulted in a AUC of 0.74 (95% CI: 0.68–0.81). An increasing predictive probability of a patient having LOS >7 days was seen with PHORS scores ≥ 2 (p < .001) (Fig 2).

### PHORS risk score and outcomes

There were 10 post-procedure deaths (mortality rate 2.3%) among all admissions in the study cohort occurring within 30 days of the procedure date, 35 (6.4%) unplanned ICU admissions during the post-procedure hospital stay, and 89 (16.2%) readmissions within 30 days of the procedure. One hundred thirty-seven cases (25.0%) were associated with one or more other complications (Table 3). A higher prevalence of any post-procedure complication was seen in patients with LOS > 7 days.

**Table 3:** Post-procedure complications by PHORS group.

Complications	Risk Score < 2 (n = 214)	Risk Score ≥2 (n = 336)	p-value
MI (%)	0 (0)	1 (0.3)	1.00
Cardiac arrest (%)	0 (0)	2 (0.6)	.52
CHF (%)	2 (1.0)	6 (1.8)	.49
ARDS (%)	2 (0.9)	2 (0.6)	1.00
Respiratory depression (%)	1 (0.5)	2 (0.6)	1.00
Reintubation (%)	1 (0.5)	6 (1.8)	.26
Stroke/TIA (%)	1 (0.5)	0 (0)	.39
VTE (%)	4 (1.9)	1 (0.3)	.08
Arrhythmia (%)	10 (4.7)	18 (5.4)	.60
Persistent hypotension (%)	3 (1.4)	7 (2.1)	.27
Acute renal failure (%)	6 (2.8)	6 (1.8)	.55
Hemorrhage requiring transfusion or re-operation (%)	2 (0.9)	6 (1.8)	.49
Hematoma (%)	2 (0.9)	0 (0)	.15
Wound debridement/revision (%)	0 (0)	10 (2.3)	.10
Re-operation for bleeding (%)	1 (0.5)	4 (1.2)	.66
Syncope (%)	1 (0.5)	2 (0.6)	1.00
Delirium (%)	5 (2.3)	12 (3.6)	.59
PNA (%)	2 (0.9)	6 (1.8)	.49
Sepsis (%)	3 (1.4)	2 (0.6)	.38
UTI (%)	1 (0.5)	2 (0.6)	1.000
SSI (%)	0 (0)	3 (0.9)	.29
Antibiotic administered for other presumed/confirmed infection (%)	4 (1.9)	6 (1.8)	1.0
Other	12 (5.6)	26 (7.7)	.39

Data reported as n (%) and representative of individual procedures. Statistical analysis adjusted for multiple admissions. Abbreviations: CHF = congestive heart failure; MI = myocardial infarction; PNA = pneumonia; SSI = surgical site infection; TIA = transient ischemic attack; UTI = urinary tract infection; VTE = venous thromboembolism

The most common complications in the study cohort were arrhythmias, acute renal failure, and delirium. Compared to patients with self-reported exercise tolerance ≥4 METs, procedures undertaken in patients with <4 METs were significantly more likely to experience a major complication (29.8% vs. 20.3%; p = .010). There were no significant differences with respect to unplanned ICU admissions (7.7% vs. 5.1%; p = .23) and hospital readmissions after discharge (19.0% vs. 13.4%; p = .08). Mortality was not significantly different between groups, with 7 deaths (2.6%) in the FS < 4 METs group, compared to 3 deaths (1.1%) in the FS ≥4 METs group (p = .10).

Procedures undertaken in patients with PHORS ≥2 demonstrated a statistically significant higher prevalence of any post-procedure complication within 30 days of the procedure (28.7% vs. 19.2%; OR 1.7, 95% CI: 1.1–2.5; p = .015). Three deaths occurred in patients with PHORS <2 (1.4%), while 9 deaths occurred in patients with PHORS ≥2 (3.5%), a difference which was not statistically significant (p = .75). There was no statistical difference in 30-day hospital readmission rates between the PHORS groups (17.6% vs 14.0%; p = .29). A statistically significant difference in unplanned ICU admissions was seen amongst procedures completed between the PHORS groups (8.6% vs. 2.8%; OR 3.3; 95% CI 1.3–8.0; p = .007). In the subgroup of intrathoracic surgical procedures, there were no significant differences in any outcome variables between the PHORS groups.

## Discussion

With improving treatments and survival, an increasing number of PHTN patients are presenting to the pre-anesthesia clinic for evaluation prior to elective surgery. Previous studies have focused on objective non-invasive alternatives such as the six-minute walk test and sub-maximal exercise stress testing to risk stratify PHTN patients. [4,10,18,30] Other studies using measured functional status as a risk stratification tool in PHTN patients confirm that poor exercise tolerance is associated with higher-risk patients. [4,18] No previous studies have examined patient self-reported exercise capacity as a predictor of post-procedure outcomes in PHTN patients. We postulated that in a heterogenous PHTN patient population with recent ECHO, further pre-procedure testing such as repeat ECHO should be targeted to higher risk patients as determined by patient and procedural factors such as self-reported exercise. Low and moderate risk patients should receive standard pre-operative evaluation, including routine assessment of the etiology for their PHTN (based on WHO classification system), prior to a decision for a repeat ECHO. In this study we found a significant association between patient self-reported excretes tolerance and LOS > 7 days. However, no ECHO measurement was independently associated with a LOS > 7 days in the multiple regression.

As a practical matter, initial pre-operative evaluation in the pre-anesthesia clinic generally precedes evaluation of underlying cause (e.g. WHO classification) and often determines the need for further evaluation of PHTN etiology (if no underlying etiology has been identified) and risk stratification. Thus, our study included procedures completed in patients with both pre-capillary and post-capillary PHTN, as evidenced by the significant number of patients with concurrent left-heart dysfunction. This is an important distinction to consider when targeting management and risk-stratification protocols. [24] In the current study, poor FS was also associated with decreased LVEF, but not post-capillary (WHO Class II) PHTN classification. An explanation for this disparity is that some patients could have presented with a combined pre- and post-capillary PHTN, evidenced by the fact that many patients with WHO Class I (e.g. pre-capillary) PHTN had additional cardiac risk factors that pre-disposed them to left-sided heart dysfunction. Furthermore, many patients with post-capillary PHTN have preserved EF, which could be a result of myocardial thickening, elevation of left ventricle end-diastolic pressure, and the resulting chronic hemodynamic strain on the right ventricle. [31]

Previous studies have demonstrated the value of right ventricle ECHO measurements, particularly decrements in RV strain, on morbidity and mortality in nonsurgical patients. [32–40] Sachdev et al. demonstrated that a greater depression in RV strain in patients with primary PAH was predictive of mortality up to 4 years after evaluation. [38] However, all of these studies were reported on non-surgical PHTN patients primarily carrying a diagnosis of PAH, with a focus on long-term (>1 year) outcomes. While there was a strong correlation between FS and outcomes, the addition of ECHO indicators of severe PHTN (e.g. PASP/RVSP or RAP) to a risk score model with only clinical characteristics did not improve predictive value. In reconciling the differences between these published findings and our study, it stands that post-capillary pathophysiology and left-heart dysfunction confound the predictive value of both functional status and RV parameters on outcomes. [41] However, post-capillary pathophysiology (e.g. WHO class II) was not associated with a longer LOS, and a multiple logistic regression adjusted PHTN etiologies did not demonstrate a change of predictive value for functional status or other included variables. Furthermore, poor self-reported exercise tolerance (FS <4 METs) was significantly associated with elevated RAP, a marker of severe PHTN, but not PASP, a finding that was also consistent with the analysis of the PAH subgroup. This finding suggests that FS lacked the granularity to distinguish severe PHTN from mild or moderate PHTN by PASP criteria. [42–44]

Our study found a perioperative mortality rate of 2.3% and an overall post-procedure complication rate of 25% in our PHTN patients. This is in line with previous studies of PHTN patients, which have reported a post-procedure mortality rate of 2.0% to 18.0% and a major post-procedure complication rate of 6.1% to 36% with a trend for improved mortality and morbidity rates in more recent publications. [4,6,8–10] Ramakrishna et al. demonstrated a higher perioperative cardiopulmonary complication (38%) and mortality rate (7%), primarily due to respiratory failure or right-sided heart failure. [10] In their cohort of total hip arthroplasty patients, Memtsoudis et al. reported a mortality rate of 2.4% compared to a rate of 0.6% in a matched non-PHTN control group. [6] While previous perioperative studies on PHTN patients primarily focus on those with WHO Group I diagnosis (pulmonary arterial hypertension), our current study evaluates the mortality rate in patients with any PHTN etiology, including left ventricle dysfunction (WHO Group II), intrinsic lung disease (WHO Group III) and a history of thromboembolic disease (WHO Group IV). [45] Given the preponderance of patients with post-capillary pathophysiology or decreased LVEF, one must consider the outcomes in the context of the cardiac and end-organ dysfunction that occurs more frequently than in patients with pre-capillary PHTN (i.e. primary pulmonary arterial hypertension).

Similar to the Revised Cardiac Risk Index (RCRI) employed in the ACC/AHA guidelines, [27] we utilized the independent predictors of longer LOS to construct a risk stratification system (PHORS) and apply it to our assessment of mortality and morbidity in the study population. We demonstrate that FS <4 METs, open surgical approach, procedure duration, ASA class and absence of systemic hypertension, but not post-capillary PHTN, were independent significant predictors of an increased likelihood of LOS >7 days. Ramakrishna et al. conducted a similar analysis in their experience with 145 patients with PAH (WHO Class I), yielding NYHA class, high-risk surgery and procedural duration as independently predictive variables of post-procedure morbidity. [10] During construction of the PHORS model, we deliberately excluded ASA classification, which has significant overlap with self-reported FS. A lower proportion of patients with LOS >7 days had systemic hypertension, which is in contrast with the findings in previous studies. [10,46] This might be explained by our inclusion of PHTN patients with left-sided cardiac dysfunction (WHO Group II), who are more likely to have lower blood pressure owing to systolic (i.e. cardiogenic) dysfunction. Renal dysfunction (serum creatinine > 1.5 mg/dl), which has been demonstrated to prognosticate post-procedure morbidities and mortality in certain surgeries, [47] was not significantly associated with outcome in our study which focused on a multitude of procedure types.

We present the first model using a set of well-established, internally validated, and statistically significant variables that can be easily elicited from the patient’s medical history and planned surgical approach. A PHORS ≥2 predicted a significant greater probability of complications, although the absolute number of mortalities was quite low in this study. A finding that a patient has a PHORS score ≥2 might, for example trigger the need for further evaluation of LV versus RV function as a means of pursuing further optimization of the patient, whereas a low risk score (<2) could indicate that further evaluation is unlikely to further lower risk despite significantly increased expense. However, we acknowledge the importance of including previous ECHO data and underlying etiology (based on WHO classification and pre- vs. post-capillary pathophysiology) to improve risk stratification over and above that of the PHORS risk stratification system. In every case, the pre-operative clinician should thoroughly assess the underlying cause of PHTN to further optimize the patient prior to surgery. For example, a patient with WHO Class II PHTN would benefit from continued management of left-sided cardiac dysfunction or stenotic valvular lesions, while a patient with WHO Class IV PHTN may have underlying thromboembolic disease that requires chronic anticoagulation. Based on this assessment, continued treatments to optimize the patient prior to surgery may require additional ECHO assessment to monitor improvements in parameters related to PHTN.

We acknowledge limitations with our retrospective study, notably that patients with PHTN due to left-sided cardiac dysfunction were included. Although our study did not find an association between pre- versus post-capillary pathophysiology and outcomes, it is conceivable that many patients could have developed a combined (e.g. pre-capillary and post-capillary) PHTN pathophysiology since the time of their original diagnosis. In these patients, it is difficult to discern whether a decrement in functional status is due to a dysfunctional LV state (e.g. CAD with or without prior intervention, CHF), chronic lung disease (e.g. asthma, COPD) or PHTN itself. Next, we did not include a matched case control cohort of non-PHTN patients, and thus cannot make conclusions about outcome differences between patients with and without PHTN. Rather, our study was intended to enable the perioperative physician to discriminate outcomes between low and high-risk PHORS group PHTN patients, potentially allowing the perioperative physician to determine which patients may need further perioperative testing. In addition, our study cohort included very few patients with severe PHTN (PASP ≥ 59 mmHg), likely due to the lower prevalence of severe PHTN and the tendency to avoid elective surgery in such high-risk patients. Nevertheless, our study population was much larger than previously reported literature. Finally, we excluded intraoperative complications from the risk scoring model, which is intended to identify pre-procedure factors that can be recognized by the healthcare team.

## Conclusions

Self-reported functional status represents a simple, cost-free risk stratification tool in a real-world population of PHTN patients. A risk assessment model (PHORS), in conjunction with the surgeon team and a PHTN specialist, can identify higher risk PHTN patients who might reasonably need to undergo further testing, including repeat ECHO, and optimization prior to elective surgery. A thorough evaluation of underlying etiologies of PHTN should be undertaken in every patient.

## Supporting information

S1 Table Demographic characteristics of patients with PHTN by LOS. (DOCX)

S2 Table Procedure categories of study population by LOS. (DOCX)

S3 Table Intraoperative characteristics of patients with PHTN by LOS. (DOCX)

## Abbreviations

ASA: American Society of Anesthesiologists
CAD: coronary artery disease
CHF: congestive heart failure
COPD: chronic obstructive pulmonary disease
ECHO: echocardiography
FS: functional status
LOS: length of stay
METS: metabolic equivalents of task
NCS: noncardiac surgery
PAH: pulmonary arterial hypertension
PASP: pulmonary artery systolic pressure
PHTN: pulmonary hypertension
RV: right ventricle
VTE: venous thromboembolism
