# Supplementary material for: Self-reported functional status predicts post-operative outcomes in non-cardiac surgery patients with pulmonary hypertension
Source: PLoS One. 2018 Aug 16;13(8):e0201914. doi: 10.1371/journal.pone.0201914 (PMC6095504; doi:10.1371/journal.pone.0201914)
Supplement: S1 Table — (DOCX) [file pone.0201914.s001.docx]

Supplemental Table 1**:** Demographic characteristics of patients with PHTN by LOS status

| **Characteristics** | **LOS** ≤ **7 days**  **(n = 433)** | **LOS > 7 days**  **(n = 117)** | **p-value** |
| --- | --- | --- | --- |
| Male (%) | 229 (53) | 69 (59) | .25 |
| Age (years) (mean [SD]) | 60 (14) | 60 (16) | .85 |
| Body mass (kg) (mean [SD]) | 92 (33) | 89 (34) | .45 |
| Height (cm) (mean [SD]) | 170 (10) | 171 (10) | .26 |
| BMI (kg/m^2^) (mean [SD]) | 32 (12) | 31 (12) | .29 |
| Anticipated inpatient post-operative disposition | 248 (57) | 117 (100) | <.001 |
| ASA classification (%)  II  III  IV | 39 (9)  311(72)  83 (19) | 4 (3)  66 (56)  47 (40) | <.001 |
| WHO Class (%)  I  II  III  IV  V | 251 (58)  102 (24)  59 (14)  19 (4)  2 (0.5) | 54 (46)  34 (29)  21 (18)  7 (6)  1 (1) | .25 |
| Post-capillary PHTN (%) | 102 (24) | 34 (29) | .23 |
| Current tobacco use (%) | 27 (6) | 9 (8) | .53 |
| Poor self-reported FS  (< 4 METs) (%) | 197 (46) | 76 (65) | <.001 |
| Open surgical approach (%) | 205 (47) | 87 (74) | <.001 |
| Systemic hypertension (%) | 298 (69) | 67 (57) | .02 |
| Angina (%) | 32 (7) | 8 (7) | 1.00 |
| Coronary artery disease (%) | 139 (32) | 44 (38) | .22 |
| Congestive heart failure (%) | 135 (31) | 40 (34) | .58 |
| Arrhythmia (%) | 191 (44) | 54 (46) | .75 |
| Venous thromboembolism (%) | 24 (6) | 10 (9) | .28 |
| Asthma (%) | 64 (15) | 14 (12) | .55 |
| COPD (%) | 53 (12) | 21 (18) | .125 |
| Obstructive sleep apnea (%) | 119 (28) | 23 (20) | .10 |
| Diabetes (%) | 130 (30) | 26 (21) | .11 |
| Renal failure  (serum creatinine > 1.5 mg/dl) (%)* | 71 (23) | 31 (30) | .20 |
| PHTN medical therapy (%) | 13 (3) | 4 (3) | .77 |
| Number of procedures (n) (median [IQR)] | 2 (1 - 4) | 2 (1 - 4) | .73 |
| Most recent procedure (%) | 298 (69) | 72 (62) | .15 |
| Procedure length (hours)  (median [IQR)]** | 2.2 (0.9 - 4.0) | 1.5 (0.6 - 2.6) | <.001 |
| **ECHO Finding** | | | |
| RAP ≥ 10 mmHg*** | 130 (42) | 39 (44) | .72 |
| PASP or RVSP  (median [IQR)]**** | 44 (32 - 55) | 46 (33 - 59) | .15 |
| PASP > 59mmHg**** | 37 (9) | 13 (11) | .47 |
| LVEF < 40%***** | 125 (29) | 38 (34) | .36 |

Data reported as n (%) unless otherwise specified. P-values obtained from univariate logistic regression analysis after adjustment for multiple admissions.

* Data available in n = 306 cases (LOS ≤ 7 days) and n = 105 cases (LOS > 7 days)

** Data available in n = 431 cases (LOS ≤ 7 days) and n = 116 cases (LOS > 7 days)

*** Data available in n = 313 cases (LOS ≤ 7 days) and n = 89 cases (LOS > 7 days)
**** Data (PASP or RVSP) available in n = 413 cases (LOS ≤ 7 days) and n = 114 cases (LOS > 7 days).

***** Data available in n = 403 cases (LOS ≤ 7 days) and n = 102 cases (LOS > 7 days)

Abbreviations: ASA = American Society of Anesthesiologists; BMI = body mass index; COPD = chronic obstructive pulmonary disease; ECHO = echocardiography; FS = functional status; IQR = interquartile range; PAH = pulmonary arterial hypertension; PASP = pulmonary artery systolic pressure; PHTN = pulmonary hypertension; RAP = right atrial pressure; RVSP = right ventricular systolic pressure; WHO = World Health Organization
