# Supplementary material for: Self-reported functional status predicts post-operative outcomes in non-cardiac surgery patients with pulmonary hypertension
Source: PLoS One. 2018 Aug 16;13(8):e0201914. doi: 10.1371/journal.pone.0201914 (PMC6095504; doi:10.1371/journal.pone.0201914)
Supplement: S2 Table — (DOCX) [file pone.0201914.s002.docx]

Supplemental Table 2**:** Procedure categories of study population by LOS

| **Procedure category** | **LOS** ≤ **7 days**  **(n = 433)** | **LOS > 7 days**  **(n = 117)** | **p-value** |
| --- | --- | --- | --- |
| HIGH RISK Procedures | | | |
| Intra-abdominal (%) | 82 (19.4) | 32 (27.4) | .38 |
| Intra-thoracic (%) | 14 (3.3) | 17 (14.5) | .002 |
| Suprainguinal Vascular (%) | 18 (4.3) | 2 (1.7) | .039 |
| Intracranial (%) | 5 (1.2) | 1 (0.9) | .67 |
|  |  |  |  |
| NON-HIGH RISK Procedures | | | |
| Plastics – breast (%) | 17 (4.0) | 1 (0.9) | -- |
| Cardiology (catheterization, electrophysiology study, transesophageal ECHO) (%) | 83 (19.7) | 21 (17.9) | -- |
| Thoracic (e.g. bronchoscopy, mediastinoscopy) (%) | 9 (2.1) | 2 (1.7) | -- |
| Peripheral vascular (%) | 5 (1.2) | 9 (7.7) | -- |
| Otolaryngology (%) | 35 (8.3) | 9 (7.7) | -- |
| Dental (%) | 3 (0.7) | 3 (2.6) | -- |
| Gastroenterology (%) | 48 (11.4) | 3 (2.6) | -- |
| Gynecology (%) | 12 (2.8) | 3 (2.6) | -- |
| Interventional radiology (%) | 6 (1.4) | 2 (1.7) | -- |
| Orthopedic – hip (%) | 3 (0.7) | 1 (0.9) | -- |
| Orthopedic – extremity (%) | 30 (7.1) | 4 (3.4) | -- |
| Orthopedic – spine (%) | 16 (3.8) | 3 (2.6) | -- |
| Plastics – other (%) | 7 (1.7) | 2 (1.7) | -- |
| Urology (%) | 36 (8.5) | 2 (1.7) | -- |
| Other | 4 (0.9) | 0 (0) | -- |

Data reported as n (%) unless otherwise specified.
