# Supplementary material for: Self-reported functional status predicts post-operative outcomes in non-cardiac surgery patients with pulmonary hypertension
Source: PLoS One. 2018 Aug 16;13(8):e0201914. doi: 10.1371/journal.pone.0201914 (PMC6095504; doi:10.1371/journal.pone.0201914)
Supplement: S3 Table — (DOCX) [file pone.0201914.s003.docx]

Supplemental Table 3**:** Intra-procedure characteristics of patients with PHTN by LOS status

| **Characteristics** | **LOS** ≤ **7 days**  **(n = 433)** | **LOS > 7 days**  **(n = 117)** | **p-value** |
| --- | --- | --- | --- |
| Cardiac arrest | 0 (0) | 0 (0) | -- |
| Vasopressor use  Dobutamine  Epinephrine  Norepinephrine  Phenylephrine  Vasopressin | 1 (0.2)  4 (0.9)  1 (0.2)  186 (43.3)  49 (12.1) | 3 (2.6)  6 (5.1)  1 (0.9)  64 (55.2)  29 (25.9) | .032  .008  .38  .027  .001 |
| Inhaled nitric oxide (NO)*** | 7 (1.6) | 2 (1.7) | 1.00 |
| Post-procedure ventilatory support | 5 (1.2) | 7 (6.0) | .005 |

Data reported as n (%) unless otherwise specified.
